# ^18^F-APN-1607 Tau Positron Emission Tomography Imaging for Evaluating Disease Progression in Alzheimer’s Disease

**DOI:** 10.3389/fnagi.2021.789054

**Published:** 2022-02-10

**Authors:** Xiaojun Xu, Weiwei Ruan, Fang Liu, Yongkang Gai, Qingyao Liu, Ying Su, Zhihou Liang, Xun Sun, Xiaoli Lan

**Affiliations:** ^1^Department of Nuclear Medicine, Union Hospital, Tongji Medical College, Huazhong University of Science and Technology, Wuhan, China; ^2^Hubei Province Key Laboratory of Molecular Imaging, Wuhan, China; ^3^Department of Neurology, Union Hospital, Tongji Medical College, Huazhong University of Science and Technology, Wuhan, China

**Keywords:** Alzheimer’s disease, ^18^F-APN-1607, tau, positron emission tomography, progression

## Abstract

**Purpose:**

^18^F-APN-1607 is a novel tau positron emission tomography (PET) tracer characterized with high binding affinity for 3− and 4-repeat tau deposits. The aim was to analyze the spatial distribution of ^18^F-APN-1607 PET imaging in Alzheimer’s disease (AD) subjects with different stages and to investigate the relationship between the change of tau deposition and overall disease progression.

**Methods:**

We retrospectively analyzed the ^18^F-APN-1607 PET imaging of 31 subjects with clinically and imaging defined as AD. According to the Mini-Mental State Examination (MMSE) score, patients were divided into three groups, namely, mild (≥21, *n* = 7), moderate (10–20, *n* = 16), and severe (≤9, *n* = 8). PET imaging was segmented to 70 regions of interest (ROIs) and extracted the standard uptake value (SUV) of each ROI. SUV ratio (SUVR) was calculated from the ratio of SUV in different brain regions to the cerebellar cortex. The regions were defined as positive and negative with unsupervised cluster analysis according to SUVR. The SUVRs of each region were compared among groups with the one-way ANOVA or Kruskal–Wallis *H* test. Furthermore, the correlations between MMSE score and regional SUVR were calculated with Pearson or Spearman correlation analysis.

**Results:**

There were no significant differences among groups in gender (χ^2^ = 3.814, *P* = 0.161), age of onset (*P* = 0.170), age (*P* = 0.109), and education level (*P* = 0.065). With the disease progression, the ^18^F-APN-1607 PET imaging showed the spread of tau deposition from the hippocampus, posterior cingulate gyrus (PCG), and lateral temporal cortex (LTC) to the parietal and occipital lobes, and finally to the frontal lobe. Between the mild and moderate groups, the main brain areas with significant differences in ^18^F-APN-1607 uptake were supplementary motor area (SMA), cuneus, precuneus, occipital lobule, paracentral lobule, right angular gyrus, and parietal, which could be used for early disease progression assessment (*P* < 0.05). There were significant differences in the frontal lobe, right temporal lobe, and fusiform gyrus between the moderate and severe groups, which might be suitable for the late-stage disease progression assessment (*P* < 0.05).

**Conclusion:**

^18^F-APN-1607 PET may serve as an effective imaging marker for visualizing the change pattern of tau protein deposition in AD patients, and its uptake level in certain brain regions is closely related to the severity of cognitive impairment. These indicate the potential of ^18^F-APN-1607 PET for the *in vivo* evaluation of the progression of AD.

## Introduction

Alzheimer’s disease (AD) is the most common cause of dementia in elderly people, which is mainly manifested as learning and memory impairment, aphasia, agnosia, impairment of visual and spatial skills, change in abstract thinking, and gradual impairment of daily functions (Longhe, 2020). So far, AD could not be prevented or cured, and therefore, early diagnosis and disease evaluation were critical to treatment and prognosis.

Although the details of the pathological mechanism of AD were not completely clear, the main pathological features were extracellular plaques composed of Aβ-amyloid (Aβ) and intracellular neurofibrillary tangles (NFTs) composed of hyperphosphorylated tau protein. It was generally believed that the above-mentioned pathological changes had appeared more than 10 years before the appearance of clinical symptoms (Cho et al., 2016). Autopsy studies had shown that, compared with Aβ plaque density, NFT was believed to be more directly related to neuron loss and synaptic dysfunction and therefore more closely related to AD cognitive impairment (Hadjichrysanthou et al., 2020).

The latest development of positron emission tomography (PET) imaging allowed *in vivo* visualizing and tracking of the pathophysiological changes of AD and was currently the best method for non-invasive detection of AD neuropathology (Chandra et al., 2019; Chételat et al., 2020; Villa et al., 2020). National Institute of Aging, Alzheimer’s Association (NIA-AA) had made it clear that, regardless of cognitive symptoms, AD could be determined only based on abnormal Aβ and tau protein deposition (Jack et al., 2018).

Aβ aggregation measured by amyloid PET has been confirmed to be a feasible biomarker of AD (Jack et al., 2018; Chételat et al., 2020; Jiao et al., 2021). According to available reports, the sensitivity of Aβ PET imaging was comparable to that of autopsy as the gold standard (Chételat et al., 2020; Jiao et al., 2021). However, Aβ plaques reach a plateau in the early stage of the disease, and Aβ load did not show a good correlation with the severity of symptoms and the progression of the later stage of the disease. Therefore, amyloid PET imaging alone was considered insufficient to assess the severity of AD, which limited its value in clinical practice (Koychev et al., 2017; Chételat et al., 2020).

Tau pathology had been proved to be highly related to neurodegeneration and cognitive ability decline, so it may be used as one of the biomarkers for disease severity (Cho et al., 2016; Hadjichrysanthou et al., 2020). The latest development of molecular probes targeting tau made it possible to image tau deposition with PET *in vivo* non-invasively and dynamically, which suggested a reliable method to characterize AD (Villemagne et al., 2014; Baghel et al., 2019; Teng et al., 2019; Zhao et al., 2019; Chételat et al., 2020; Cho et al., 2020). Moreover, studies had shown that tau PET uptake was positively correlated with cortical atrophy and disease severity and could reflect the pathological stage of AD (Baghel et al., 2019; Chételat et al., 2020; Cho et al., 2020).

Based on ^11^C-PBB3 tracer, the derivative ^18^F-PM-PBB3 (also known as ^18^F-APN-1607) had been synthesized and proved with better imaging properties. Both preclinical and clinical studies had proved that ^18^F-APN-1607 highly selectively combined with the pathological tau of AD and could visualize the spatial distribution of tau deposition in AD (Ni et al., 2018; Hsu et al., 2020; Weng et al., 2020; Tagai et al., 2021). In addition, it also found that ^18^F-APN-1607 uptake was closely related to the cognitive changes, and the regional uptakes of AD-related cortical areas were significantly correlated with Alzheimer’s Disease Assessment Scale-Cognitive section (ADAS-cog) score, indicating that tau deposition may be related to the clinical severity of AD (Hsu et al., 2020).

Although there are some publications on ^18^F-APN-1607 PET, as a relative novel tracer, some questions are yet to be answered. The relationship and change patterns are needed to be verified between the uptake level of ^18^F-APN-1607 in different brain regions and AD progression, which is important to understand the trajectory of the disease and find the objective evidence for judging the stage of AD. Therefore, the purpose of this study was to explore the spatial distribution of tau protein deposition with ^18^F-APN-1607 PET in different severity AD patients and in-depth dig the correlation between the regional uptake and the overall disease progression.

## Materials and Methods

### Patients

The study was approved by the Ethics Committee of Tongji Medical College, Huazhong University of Science and Technology, and registered at clinical trail.gov (NCT number 05003830). Written informed consents were obtained from all participants.

We retrospectively analyzed the ^18^F-APN-1607 PET imaging and clinical characteristics of the AD (AD-MCI or AD-Dementia) patients diagnosed in our hospital from July 2020 to July 2021. Inclusion criteria were (1) patients were diagnosed strictly in accordance with the 2011 NIA-AA diagnostic criteria (Jack et al., 2011); (2) patients confirmed as positive for β-amyloid (Aβ) deposition with PET imaging by visual assessment; and (3) patients underwent ^18^F-APN-1607 PET imaging in 2 months after the amyloid PET imaging. Exclusion criteria were (1) patients with significant cerebrovascular disease, with multiple or extensive cerebral infarction; (2) patients with dementia or cognition dysfunction due to other reasons, such as Parkinson’s disease dementia, vascular dementia, frontotemporal dementia, Lewy body dementia, and so on; (3) people with drug or alcohol abuse or dependence; (4) patients with history of epilepsy; (5) people with claustrophobia or other reasons who could not cooperate with the examination; and (6) patients who showed obvious movement during PET scanning which affected image quality and image interpretation.

### Clinical Assessment

The primary clinical parameters including the gender, age, age of onset, and education level were consulted and recorded. The cognitive function was assessed using the Mini-Mental State Examination (MMSE) score. According to the MMSE score, patients were divided into three groups: mild (≥21), moderate (10–20), and severe (≤9) (Abdullah et al., 2020). The clinical assessment was completed by two physicians (YS and ZL).

### Image Acquisition and Reconstruction

Nervous system-related drugs were stopped for more than 12 h before scanning. The precursor of APN-1607 was obtained from APRINOIA Therapeutics (Suzhou, China). According to the method described before (Weng et al., 2020), ^18^F-APN-1607 was prepared on an ^18^F-multifunction synthesizer (GE TRACERlab FxFn), and the radiochemical purity was >95%. ^18^F-APN-1607 was administrated intravenously (3.7–5.5 MBq/kg) under the green light-emitting diode light (510 nm) illumination. Resting for 60 min, the PET scanning was completed with hybrid PET/MR (3.0 T, SIGNA TOF-PET/MR, GE Healthcare). During 20 min PET acquisition, the 3D T1-weighted imaging [three-dimensional gradient echo sequence, flip angle = 12°, time of echo (TE)/time of repetition (TR) = 2.6/6.9 ms, bandwidth = 50 kHz, FOV = 24 cm × 24 cm, matrix = 384 × 384] was obtained simultaneously. The PET data were reconstructed using the ordered subsets expectation maximum (OSEM) algorithm (FOV = 30 cm × 30 cm, matrix = 192 × 192, filter cutoff = 3.0 mm, subsets = 28, iterations = 3), and the time-of-flight (TOF) technique was utilized for the reconstruction. The PET attenuation correction for PET/MR was atlas-based MRI attenuation correction, combined with Dixon water-fat separation methods.

### Data Processing

The data processing for the ^18^F-APN-1607 mainly contained two steps: the structural brain segmentation and standard uptake value (SUV) extraction for regions of interest (ROIs). SUV ratio (SUVR) was calculated from the ratio of SUV in different brain regions to the cerebellar cortex. All imaging data were converted to neuroimaging informatics technology initiative (NIfTI) format with the MRIcron tool. The 3D T1 images were normalized to the Montreal Neurological Institute (MNI) standard space. Seventy brain regions were segmented based on the atlas template from the automated anatomical labeling atlas^1^. The brain segmentation was performed on the 3D-T1 sequence, and divided into brain parenchyma, ventricle, and cerebrospinal fluid. The choroid plexus, containing in the part of the ventricle, had been screened out. Due to the simultaneous acquisition of PET and MRI, the extracted 70 regional ROIs from 3D T1 imaging could be directly transferred to binary mask and used to extract the SUV of corresponding ROIs from PET images. The detailed processing could be found in our previous study (Ruan et al., 2020). The data processing was performed by the SPM12 (see text footnote 1) and MATLAB 2020b (MathWorks, Natick, MA, United States).

### Statistical Analysis

We assumed that the SUV ratio (SUVR) of the ROIs conformed to a Gaussian distribution (Krishna and Narasimha Murty, 1999). The unsupervised clustering was performed on the SUVR of each participant and performed a 2-class *k*-means clustering to determine the positive and negative ROI (Supplementary Figure 1). In each group, one positive region was defined as ≥50% subjects with positive in the same region. In the following statistics, the difference of SUVR in every brain ROIs among the three groups (i.e., mild, moderate, and severe) was tested statistically. The method used was one-way ANOVA if the data met normality and homogeneity of variance; otherwise, the Kruskal–Wallis *H* test was used. Furthermore, the correlations between MMSE score and ^18^F-APN-1607 SUVR were analyzed for every brain ROIs with Pearson correlation analysis. *P* < 0.05 was considered a statistically significant difference. All the statistical analyses were performed with SPSS (version 21; IBM).

## Results

### Participant Characteristics

A total of 31 clinically defined probable AD subjects were included in this study. Table 1 illustrates their demographic and clinical features. On average, the participants aged 60.5 ± 7.2 years (range: 51–75 years), and 54.8% (17/31) were men. The average education level of the patients was 10.0 ± 4.3 years (range: 3–22 years). The average MMSE score was 14.5 ± 7.9 (range: 1–29).

**TABLE 1:** Demographic and clinical features of the subjects.

Characteristics	Mean ± SD	Range	MMSE groups			*P*-value
			Mild (*n* = 7)	Moderate (*n* = 16)	Severe (*n* = 8)	
**Gender**						
*Male*	17		5	6	6	0.161
*Female*	14		2	10	2	
*Age of onset*	57.58 ± 7.74	46–73	62.29 ± 8.40	56.81 ± 7.50	55.25 ± 6.45	0.170
Age of PET scan	60.45 ± 7.22	51–75	65.43 ± 7.09	59.31 ± 7.03	58.38 ± 6.46	0.109
Education (y)	9.97 ± 4.32	3–22	12.14 ± 4.14	8.25 ± 3.19	11.50 ± 5.40	0.065
MMSE score	14.52 ± 7.89	1–29	26.14 ± 1.77	14.25 ± 2.79	4.88 ± 2.36	0.000

*MMSE, Mini-Mental State Examination. Mean ± SD is indicated for continuous variables. P-value referred to the difference in clinical characteristics among the three groups.*

According to the MMSE score, patients were divided into three groups, namely, mild (≥21, *n* = 7), moderate (10–20, *n* = 16), and severe (≤9, *n* = 8). There were no statistically significant differences among different groups in gender (χ^2^ = 3.814, *P* = 0.161), age of onset (*P* = 0.170), age (*P* = 0.109), and education level (*P* = 0.065).

### Distribution of ^18^F-APN-1607 Uptake in Different Groups

**FIGURE 1:**
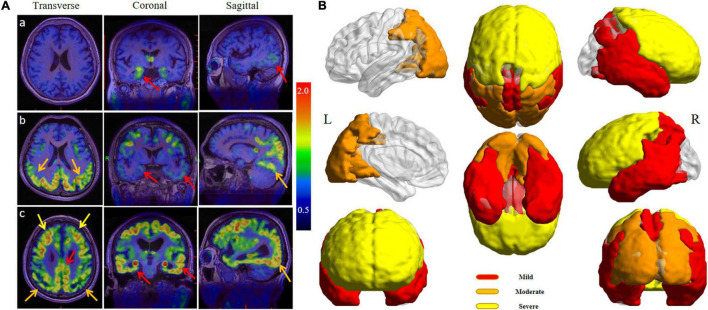
**(A)** ^18^F-APN-1607 PET/MR fusion images of three AD patients with varying degrees. **(a)** A 67-year-old woman complained of memory decline for 2 years (MMSE = 26, mild group), and the uptake of ^18^F-APN-1607 could be seen in the hippocampus and posterolateral temporal cortex (red arrows). **(b)** A 54-year-old man was with progressive memory loss for 5 years and aggravated for 2 years (MMSE = 18, moderate group), and the high uptake was expanded to the parietal and occipital lobes (orange arrows). **(c)** A 67-year-old man suffered from memory loss for 7 years and self-care disabled for 6 months (MMSE = 5, severe group), and high uptake had been spread to the whole brain including the frontal lobe (yellow arrows). **(B)** The full-view sketch map for expanding the tau deposits according to the ^18^F-APN-1607 PET. The deposition was started from the medial and lateral temporal lobes, posterior cingulate gyrus (PCG), and precuneus (red areas), then spread to the parietal and occipital cortex (orange areas), and finally affected the frontal lobe (yellow areas). L means left and R means right.

Unsupervised cluster analysis showed that AD patients had different degrees of tau deposition in multiple brain regions. The ^18^F-APN-1607-positive regions in the mild group mainly included the hippocampus, parahippocampal gyrus, amygdala, lateral temporal cortex (LTC), supramarginal gyrus (SMG), angular gyrus, posterior cingulate gyrus (PCG), and precuneus (Figure 1B, red area). In the moderate group, the positive regions expanded to the occipital lobe, cuneus, and parietal superior/inferior lobules (Figure 1B, orange area). In the severe group, except the regions mentioned ahead, the frontal lobe also became positive (Figure 1B, yellow area). Among all AD patients, the temporal lobe was the first involved, followed by the occipital, parietal, and, finally, the frontal lobe. Figure 1A shows the ^18^F-APN-1607 PET images of 3 AD subjects belonging to mild, moderate, and severe groups, respectively.

### Regional ^18^F-APN-1607 Uptake Level in Different Groups

**FIGURE 2:**
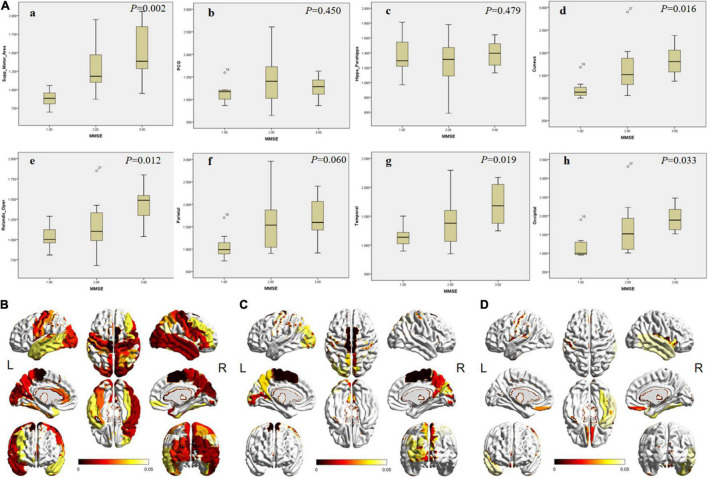
The differences of SUVR in specific brain areas among patients in different MMSE groups. **(A)** The box plot of the SUVR in specific brain areas [**(a)** SMA; **(b)** PCG; **(c)** hippocampus; **(d)** cuneus; **(e)** frontal; **(f)** parietal; **(g)** lateral temporal; and h, occipital cortex) in different groups. 1, 2, and 3 on the abscissa represent mild, moderate, and severe groups, respectively. The *P*-values among groups are shown in the upper right corner. **(B–D)** The full view of regions with significant differences in ^18^F-APN-1607 uptake. The color bar represents the *P*-value. **(B)** The overall difference among the three groups. **(C)** The difference between the mild and moderate groups. **(D)** The difference between the moderate and severe groups. SMA, supplementary motor area; PCG, posterior cingulate gyrus. L means left and R means right.

Among the three groups, except hippocampus, parahippocampal gyrus, amygdala, PCG, SMG, superial frontal gyrus (FGs), left middle and inferior frontal gyrus (FGm and FGi), and left parietal lobe, significant differences of ^18^F-APN-1607 uptake were noticed in other brain regions (Figures 2A,B and Supplementary Table 1).

Between the mild and moderate groups, significant differences could be observed in supplementary motor area (SMA), cuneus, precuneus, paracentral lobule, and left occipital lobe (Figure 2C, *P* < 0.05). The uptake was significantly different in the frontal lobe (operculum, orbital, and rectus gyrus) and right lateral temporal between the moderate and severe groups (Figure 2D, *P* < 0.05).

### Correlation Between Regional ^18^F-APN-1607 Uptake and Mini-Mental State Examination Score

**FIGURE 3:**
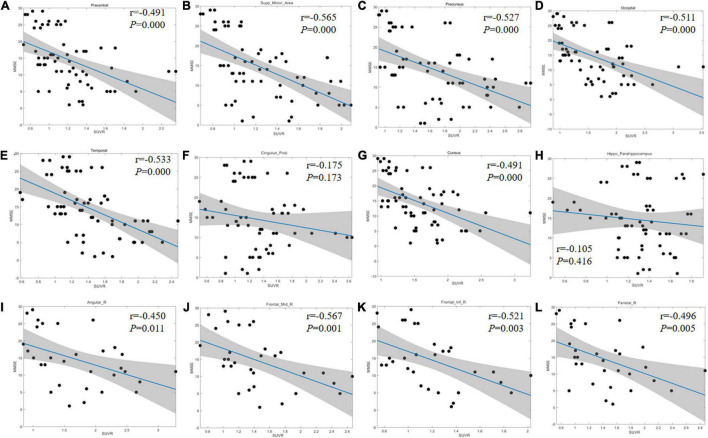
Linear regression results between MMSE score and regional SUVR. **(A–H)** Correlation coefficient graph of the bilateral precentral gyrus **(A)**, SMA **(B)**, precuneus **(C)**, occipital lobe **(D)**, lateral temporal lobe **(E)**, PCG **(F)**, cuneus **(G)**, and hippocampus **(H)**. **(I–L)** Correlation coefficient graph of the right side of angular gyrus **(I)**, FGm **(J)**, FGi **(K)**, and parietal lobe **(L)**. The gray area shows the 95% confidence interval area. The blue solid lines refer to the mean fitting curves. SMA, supplementary motor area; PCG, posterior cingulate gyrus; FGm, middle frontal gyrus; FGi, inferior frontal gyrus.

In the regions with significant difference among groups, the linear regression method was applied to analyze the correlation between MMSE score and regional SUVR. As the inter-group difference and correlation were similar in both sides of the precentral gyrus, SMA, PCG, precuneus, cuneus, hippocampus, lateral temporal lobe, and occipital lobe, the scatter diagrams were displayed including bilateral of the corresponding regions. For the frontal lobe (middle and inferior gyrus), angular gyrus, and parietal lobe, the scatter diagrams were displayed only for the right side because the inter-group difference was discovered to be unilateral. There was no significant correlation between the MMSE and SUVR in PCG and hippocampus (*r* = −0.175, *P* = 0.173; *r* = −0.105, *P* = 0.416). The other regions showed a strong negative correlation (*r* range: −0.401∼−0.631, *P* < 0.05; Figure 3 and Supplementary Table 1).

## Discussion

^18^F-APN-1607 was a novel PET tracer targeting tau protein which is one of the important factors for AD pathological changes. Our results showed that with the disease progression, the ^18^F-APN-1607 PET imaging showed the spread of tau deposition from the hippocampus, posterior cingulate gyrus (PCG), and lateral temporal cortex (LTC) to the parietal and occipital lobes, and finally to the frontal lobe. Uptake levels of ^18^F-APN-1607 in some brain areas may help to assess the degrees of AD progression, including supplementary motor area (SMA), cuneus, precuneus, occipital lobule, paracentral lobule, right angular gyrus, and parietal for early-stage evaluation; and frontal lobe, right temporal lobe, and fusiform gyrus for AD late-stage progression. These suggested that ^18^F-APN-1607 PET may serve as an effective imaging marker for visualizing the change of tau protein deposition in AD patients, and its uptake pattern in different brain regions may relate to the progress of cognitive impairment.

According to the Braak staging of AD-related neurofibrillary changes (Braak and Braak, 1995; Braak et al., 2006), six stages of disease propagation could be distinguished with respect to the location of the tangle-bearing neurons and the severity of changes (transentorhinal stages I–II: clinically silent cases; limbic stages III–IV: incipient AD; neocortical stages V–VI: fully developed AD). This stage pattern was derived from the histopathological staining at autopsy. Then, tau tracer could be used to describe the spatiotemporal pattern of tau deposition in AD patients, which had been replicated by tau-PET imaging studies *in vivo* (Koychev et al., 2017; Baghel et al., 2019; Das et al., 2019; Zhao et al., 2019; Cho et al., 2020; Hsu et al., 2021; Seemiller et al., 2021). In our study, 31 AD patients were divided into 3 groups (i.e., mild, moderate, and severe) according to the MMSE score. ^18^F-APN-1607 PET imaging revealed that the temporal lobe area (hippocampus, parahippocampal gyrus, amygdala, and LTC), PCG, precuneus, SMG, and angular gyrus were the most affected regions in the mild group. Then, in the moderate group, the high uptake was expanded to the occipital lobe, cuneus, and parietal superior/inferior lobules. In the severe group, the frontal lobe also became positive. This topographical progression of tau distribution was generally consistent with the Braak staging system, which means the ^18^F-APN-1607 PET imaging could be applied for visualizing disease progression in AD patients. In recent researches, 4 distinct spatiotemporal trajectories of tau pathology were identified in AD, including previously described limbic-predominant and medial temporal lobe-sparing patterns, while also discovering posterior and lateral temporal patterns resembling atypical clinical variants of AD (Vogel et al., 2021). In our study, the affected areas in the early stage were wider than those reported, which may be due to the mixture of the different subtypes and different standards to divide the stage.

Alzheimer’s disease was considered as a continuum (Jack et al., 2011), in which different regions presented different deposition models. Which region could be conducted to evaluate the disease progression? To answer this question, the SUVRs of each region were compared among different groups in our study, and many regions possessed significant differences. Furthermore, a good negative correlation between MMSE score and regional SUVR was also presented (*r* range: −0.401 to −0.631, *P* < 0.05). It was similar to the earlier reported results (Koychev et al., 2017; Baghel et al., 2019; Zhao et al., 2019; Ossenkoppele et al., 2021). Pontecorvo et al. (2017) reported that in Aβ-positive AD patients, neocortical tau tracer retention increased with the severity of the diagnosis. In the study by Hsu et al. (2020), the increased pattern of ^18^F-APN-1607 tau deposition was also proved regional, and the SUVR was significantly positively correlated with ADAS-cog and Clinical Dementia Rating Scale Sum of Boxes (CDR-SB) score in the frontal lobe, parietal lobe, temporal lobe, occipital lobe, anterior cingulate gyrus, posterior cingulate gyrus, precuneus, and parahippocampus (*P* < 0.01). These findings and the results from our study suggested that ^18^F-APN-1607 PET imaging could be served as an effective imaging marker to evaluate the disease progression.

In this study, we found that the change, deposition area, and extension of tau protein deposition in different brain regions may be in accordance with the aggravation of the disease. In the early stage, positive tau expression may be found in the medial temporal lobe area (hippocampus, parahippocampal gyrus, and amygdala) and PCG. However, the SUVR did not increase further along with the decline of MMSE score, and no statistical difference was found among the three groups (*P* > 0.05). In the previous report (Hsu et al., 2020), there was also no significant correlation between the hippocampus and ADAS-cog score (*P* = 0.53). Then, positive tau deposition could be seen in the lateral temporal lobe presenting as the continual uptake increasing from the early stage to the advanced stage. Next, positive findings appeared in parietal lobe, occipital lobe, cuneus, and precuneus, experiencing a significant increasing trend of SUVR. Notably, the deposition of tau in the frontal region may indicate the middle-late stages.

In our study, although the change modes of tracer uptake were different in various brain regions, the trends were nearly consistent with the Braak pathological pattern, in which tau aggregates spread from the medial temporal lobe and build up in larger quantities throughout the neocortex (Braak and Braak, 1995). Pathological tau might undergo cell-to-cell transmission, resulting in the transformation of normal tau in the recipient cell into misfolded tau and the formation of tau aggregates (Braak and Del Tredici, 2016; Peng et al., 2020). The ^18^F-APN-1607 PET might reveal the detailed pattern of tau deposition and the pathological progression in AD patients. The changes of the uptake in different brain regions might provide the information to evaluate the staging and clinical manifestation. The medial temporal lobe area and PCG, which presented positive in the early stage but without further increase, might serve as the marker just for early diagnosis. In the lateral temporal lobe, the uptake was continuously increased, which might be an area to monitor the overall progression. Considering the significant change during certain stage, the parietal or occipital lobe might be a monitoring site for the fast progress in the early stage, while the high uptake in the frontal lobe might be a marker for the advanced stage.

The current research still had certain limitations. First, the sample size was relatively small, which might limit the interpretation of the results and need to be expanded in the future study. Second, as a relatively new tracer, compared with other tau tracers, the sensitivity of ^18^F-APN-1607 in detecting tau deposition for early-stage AD was uncertain. In addition, although the MMSE score was a useful screening tool to evaluate the cognitive function (Folstein et al., 1975; Zhao et al., 2019; Jiao et al., 2020), it had limited value in assessing the severity of AD, and more cognitive tests are required for evaluation. Future researches should focus on the prospective longitudinal study of ^18^F-APN-1607 PET imaging, supplemented by other biomarkers and more clinical information, which may provide further reliable evidence for the application of tau PET in AD.

## Conclusion

This study showed that ^18^F-APN-1607 PET imaging could help to clarify the spatial distribution of tau deposition in AD patients and presented a negative correlation between ^18^F-APN-1607 uptake in specific brain regions and overall severity of cognitive impairment. In summary, ^18^F-APN-1607 PET may be an effective imaging marker for visualization and tracking of tau protein spreading in the brain of AD patients and possessed the potential to evaluate the disease progression of AD.

## Data Availability Statement

The original contributions presented in the study are included in the article/Supplementary Material, further inquiries can be directed to the corresponding author/s.

## Ethics Statement

The studies involving human participants were reviewed and approved by the Ethics Committee of Tongji Medical College, Huazhong University of Science and Technology. The participants provided their written informed consent to participate in this study.

## Author Contributions

XL substantially contributed to the conception and design, and revised the manuscript critically for important intellectual content. XS interpreted the data and revised the manuscript critically for important intellectual content. XX acquired, analyzed, and interpreted most of the data and drafted the article. WR acquired the PET images and analyzed some data. QL and YG prepared the compound ^18^F-APN-1607. YS and ZL provided the clinical information and made the diagnosis. All authors read and approved the final manuscript.

## Conflict of Interest

The authors declare that the research was conducted in the absence of any commercial or financial relationships that could be construed as a potential conflict of interest.

## Publisher’s Note

All claims expressed in this article are solely those of the authors and do not necessarily represent those of their affiliated organizations, or those of the publisher, the editors and the reviewers. Any product that may be evaluated in this article, or claim that may be made by its manufacturer, is not guaranteed or endorsed by the publisher.

## Footnotes

1
http://www.gin.cnrs.fr/en/tools/aal-aal2/
