# Supplementary material for: 18F-APN-1607 Tau Positron Emission Tomography Imaging for Evaluating Disease Progression in Alzheimer’s Disease
Source: Front Aging Neurosci. 2022 Feb 10;13:789054. doi: 10.3389/fnagi.2021.789054 (PMC8868571; doi:10.3389/fnagi.2021.789054)
Supplement: Supplementary file 1 [file Data_Sheet_1.docx]

**Supplementary materials.**

**Title:** ^18^F-APN-1607 Tau PET imaging for evaluating disease progression in Alzheimer’s disease

**Authors:** Xiaojun Xu^1,2^, Weiwei Ruan^1,2^, Fang Liu ^1,2^, Yongkang Gai ^1,2^, Qingyao Liu ^1,2^ Ying Su ^3^, Zhihou Liang ^3^, Xun Sun ^1,2*^, Xiaoli Lan ^1,2*^

**Affiliations:**

^1^Department of Nuclear Medicine, Union Hospital, Tongji Medical College, Huazhong University of Science and Technology, Wuhan 430022, China

^2^ Hubei Province Key Laboratory of Molecular Imaging, Wuhan 430022, China

^3^ Departments of Neurology, Union Hospital, Medical College, Huazhong University of Science and Technology, Wuhan 430022, China

***Corresponding author:**

Dr. Xiaoli Lan, M.D, Ph.D, ([xiaoli_lan@hust.edu.cn](mailto:xiaoli_lan@hust.edu.cn));

Dr. Xun Sun, M.D, Ph.D, ([xun_sun@hust.edu.cn](mailto:xun_sun@hust.edu.cn))

**Unsupervised clustering.**

Let $X=\{x_{1},x_{2},\ldots,x_{n} \}$ be the value of the SUVR, $C_{1}$ and $C_{2}$ be the clustering centers of positive and negative groups and $D_{ij}$ be the distance between the $i$-th ROI and $j$-th center. Denote $M$ be the membership matrix, where $M_{ij}$=1 represent i-th ROI belongs the K-means optimize the following objective:

$$M=\mathrm{argmin}_{M}\sum_{i=1}^{n} \sum_{j=1}^{2} D_{ij}M_{ij}$$

$$s.t., M_{ij}\in\left\{ 0,1 \right\},\forall i,j and \sum_{j=1}^{2} M_{ij}=1,\forall i$$

Here, we utilize the Euclidean Distance to calculate the distance matirx $D.$ the entire algorithm by K-means can be easily solved by EM algorithm, the algorithm can be given as follows:

| Input $X$  Output *M* |
| --- |
| Initilize $C_{1}$ and $C_{2}$  while not converge  Update *D* by Euclidean Distance;  Updata *M* by greedy research;  Update $C_{1}$ and $C_{2}$;  end  Return *M.* |


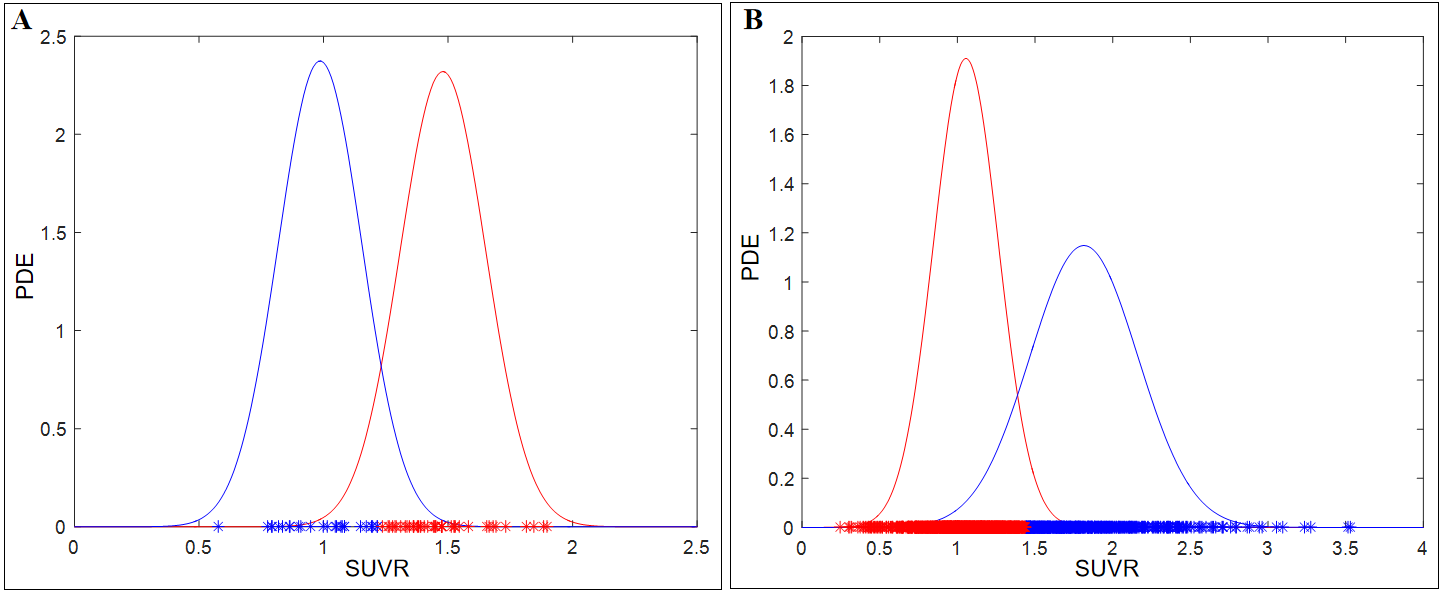


Figure S1. We assumed that the SUVR of the ROIs conformed to a Gaussian distribution. A. Individual threshold method: For each participant, we first computed all the SUVR and perform a 2-class k-means clustering to determine the positive ROIs; which was the result for one of the participants. B. Global threshold method: taking all 31 subjects as a whole, calculated the SUVR of all ROIs and obtained a cut-off value to divide the positive and negative region. PDE, Probability Density Estimation.

**Table S1.** Brain regions with significant differences in ^18^F-APN-1607 uptake and the correlation coefficient with the severity of cognitive impairment.

| **Brain regions** | **MMSE groups** | | | ***P* value (among 3 group)** | ***P* value (between two groups)** | | | **Correlation analysis** | |  |
| --- | --- | --- | --- | --- | --- | --- | --- | --- | --- | --- |
|  | ^1^Mild | ^2^Moderate | ^3^Severe |  | **1 vs 2** | **1 vs 3** | **2 vs 3** | ***r*** | ***P value*** | |
| Precentral_L | 0.94±0.16 | 1.25±0.39 | 1.46±0.34 | 0.020 | 0.055 | 0.006 | 0.152 | -0.525 | 0.002 | |
| Precentral_R | 0.96±0.11 | 1.21±0.36 | 1.35±0.23 | 0.007 | 0.067 | 0.015 | 0.282 | -0.525 | 0.002 | |
| Frontale  operculum_L | 1.05±0.17 | 1.16±.030 | 1.48±0.26 | 0.010 | 0.368 | 0.005 | 0.011 | -0.575 | 0.001 | |
| Frontale  operculum_R | 1.02±0.15 | 1.14±0.30 | 1.40±0.25 | 0.026 | 0.306 | 0.010 | 0.034 | -0.535 | 0.002 | |
| Supplementary motor area_L | 0.88±0.14 | 1.33±0.33 | 1.60±0.36 | 0.000 | 0.003 | 0.000 | 0.055 | -0.631 | 0.000 | |
| Supplementary motor area_R | 0.88±0.12 | 1.24±0.31 | 1.41±0.42 | 0.002 | 0.002 | 0.026 | 0.708 | -0.567 | 0.001 | |
| Middle frontal gyrus_R | 1.11±0.22 | 1.46±0.52 | 1.75±0.50 | 0.041 | 0.109 | 0.012 | 0.154 | -0.567 | 0.001 | |
| Inferior frontal gyrus_R | 0.94±0.14 | 1.19±0.35 | 1.42±0.27 | 0.016 | 0.068 | 0.004 | 0.096 | -0.521 | 0.003 | |
| Anterior cingulate_L | 1.00±0.12 | 1.12±0.28 | 1.31±0.18 | 0.026 | 0.422 | 0.006 | 0.174 | -0.534 | 0.002 | |
| Calcarine fissure_L | 1.11±0.18 | 1.36±0.25 | 1.45±0.15 | 0.013 | 0.017 | 0.004 | 0.310 | -0.499 | 0.003 | |
| Cuneus_L | 1.21±0.27 | 1.60±0.45 | 1.87±0.28 | 0.008 | 0.052 | 0.001 | 0.264 | -0.556 | 0.001 | |
| Cuneus_R | 1.19±0.22 | 1.60±0.52 | 1.79±0.42 | 0.009 | 0.039 | 0.014 | 0.730 | -0.456 | 0.010 | |
| Occipital_L | 1.18±0.36 | 1.65±0.59 | 1.92±0.29 | 0.022 | 0.043 | 0.006 | 0.205 | -0.600 | 0.000 | |
| Occipital_R | 1.20±0.34 | 1.58±0.65 | 1.91±0.40 | 0.011 | 0.236 | 0.008 | 0.356 | -0.577 | 0.001 | |
| Fusiform gyrus_L | 1.22±0.19 | 1.45±0.38 | 1.78±0.31 | 0.026 | 0.185 | 0.003 | 0.106 | -0.574 | 0.001 | |
| Fusiform gyrus_R | 1.24±0.19 | 1.39±0.47 | 1.75±0.30 | 0.041 | 0.396 | 0.017 | 0.042 | -0.486 | 0.003 | |
| Postcentral_L | 0.94±0.17 | 1.11±0.29 | 1.31±0.22 | 0.030 | 0.132 | 0.009 | 0.091 | -0.539 | 0.000 | |
| Postcentral_R | 0.97±0.15 | 1.19±0.27 | 1.31±0.27 | 0.021 | 0.060 | 0.030 | 0.663 | -0.495 | 0.005 | |
| Angular_R | 1.22±0.36 | 1.80±0.71 | 1.93±0.53 | 0.047 | 0.051 | 0.029 | 0.947 | -0.450 | 0.011 | |
| Precuneus_L | 1.20±0.32 | 1.73±0.58 | 1.87±0.42 | 0.033 | 0.035 | 0.013 | 0.875 | -0.549 | 0.001 | |
| Precuneus_R | 1.17±0.30 | 1.73±0.58 | 1.85±0.51 | 0.034 | 0.019 | 0.024 | 0.937 | -0.511 | 0.003 | |
| Paracentral lobule_L | 0.88±0.10 | 1.15±0.22 | 1.24±0.25 | 0.007 | 0.002 | 0.012 | 0.754 | -0.499 | 0.004 | |
| Paracentral lobule_R | 0.90±0.09 | 1.19±0.26 | 1.21±0.32 | 0.035 | 0.002 | 0.084 | 0.999 | -0.401 | 0.025 | |
| Heschl_L | 1.05±0.17 | 1.23±0.31 | 1.38±0.22 | 0.042 | 0.255 | 0.019 | 0.474 | -0.575 | 0.001 | |
| Parietal_R | 1.03±0.31 | 1.48±0.52 | 1.62±0.47 | 0.032 | 0.049 | 0.036 | 0.880 | -0.496 | 0.005 | |
| Temporal_L | 1.12±0.21 | 1.36±0.45 | 1.65±0.38 | 0.042 | 0.191 | 0.013 | 0.089 | -0.525 | 0.002 | |
| Temporal_R | 1.17±0.21 | 1.43±0.40 | 1.75±0.36 | 0.013 | 0.118 | 0.004 | 0.047 | -0.548 | 0.001 | |

Note— _L refers to the left; and _R refers to the right.

r was the Pearson or Spearman correlation coefficient.
